# Butyl 3-oxo-2,3-dihydro­benzo[*d*][1,2]thia­zole-2-carboxyl­ate

**DOI:** 10.1107/S160053681104791X

**Published:** 2011-11-23

**Authors:** Jian-Xin Yang, Xiang-Hui Wang, Xue-Mei Tan, Yin Wang, Qiang Lin

**Affiliations:** a Institute of Materials and Chemical Engineering, Hainan University, Haikou 570228, People’s Republic of China; b Institute of Environmental Science and Engineering, Kunming University of Science and Technology, Kunming 650093, People’s Republic of China; c Hainan Provincial Fine Chemical Engineering Center, Hainan University, Haikou 570228, People’s Republic of China; d College of Chemistry and Chemical Engineering, Hainan Normal University, Haikou 571100, People’s Republic of China

## Abstract

The title compound, C_12_H_13_NO_3_S, was synthesised by the reaction of benzo[*d*]isothia­zol-3(2*H*)-one with butyl alcohol in toluene. The benzoisothia­zolone ring system is almost planar with a mean deviation of 0.041 (1) Å. In the crystal, mol­ecules are linked by weak inter­molecular C—H⋯O hydrogen bonds.

## Related literature

For background to the sythesis of benzoisothia­zolone deriv­atives, see: Davis (1972 ▶); Elgazwy & Abdel-Sattar (2003 ▶). For the biological activity of 1,2-benzoisothia­zolone derivatives, see: Taubert *et al.* (2002 ▶). For structural studies of related alkyl 3-oxo-2,3-dihydro-1,2-benzothia­zole-2-carboxyl­ate derivatives, see: Wang *et al.* (2011*a* ▶,*b* ▶); Xu & Yin (2006 ▶); Cavalca *et al.* (1969 ▶).
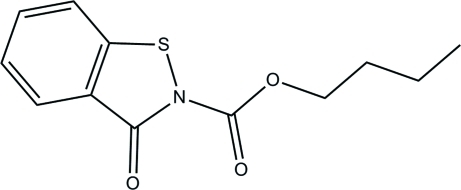


## Experimental

### 

#### Crystal data

C_12_H_13_NO_3_S
*M* *_r_* = 251.29
Monoclinic, 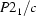

*a* = 11.730 (3) Å
*b* = 11.925 (3) Å
*c* = 8.443 (2) Å
β = 95.791 (4)°
*V* = 1175.0 (6) Å^3^
*Z* = 4
Mo *K*α radiation
μ = 0.27 mm^−1^
*T* = 153 K
0.62 × 0.36 × 0.10 mm

#### Data collection

Rigaku AFC10/Saturn724+ diffractometer
Absorption correction: multi-scan (*ABSCOR*; Higashi, 1995 ▶) *T*_min_ = 0.851, *T*_max_ = 0.973
9917 measured reflections
3092 independent reflections
2647 reflections with *I* > 2σ(*I*)
*R*_int_ = 0.028

#### Refinement

*R*[*F* ^2^ > 2σ(*F* ^2^)] = 0.036
*wR*(*F* ^2^) = 0.088
*S* = 1.00
3092 reflections
155 parameters
H-atom parameters constrained
Δρ_max_ = 0.37 e Å^−3^
Δρ_min_ = −0.19 e Å^−3^

### 

Data collection: *CrystalClear* (Rigaku, 2008 ▶); cell refinement: *CrystalClear*; data reduction: *CrystalClear*; program(s) used to solve structure: *SHELXS97* (Sheldrick, 2008 ▶); program(s) used to refine structure: *SHELXL97* (Sheldrick, 2008 ▶); molecular graphics: *SHELXTL* (Sheldrick, 2008 ▶); software used to prepare material for publication: *SHELXTL* and *publCIF* (Westrip, 2010 ▶).

## Supplementary Material

Crystal structure: contains datablock(s) I, global. DOI: 10.1107/S160053681104791X/kp2366sup1.cif

Structure factors: contains datablock(s) I. DOI: 10.1107/S160053681104791X/kp2366Isup2.hkl

Supplementary material file. DOI: 10.1107/S160053681104791X/kp2366Isup3.cml

Additional supplementary materials:  crystallographic information; 3D view; checkCIF report

## Figures and Tables

**Table 1:** Hydrogen-bond geometry (Å, °)

*D*—H⋯*A*	*D*—H	H⋯*A*	*D*⋯*A*	*D*—H⋯*A*
C2—H2⋯O1^i^	0.95	2.46	3.1610 (19)	131
C2—H2⋯O3^i^	0.95	2.39	3.2987 (18)	159

Symmetry code: (i) 
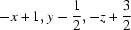
.

**Fig. 1.**
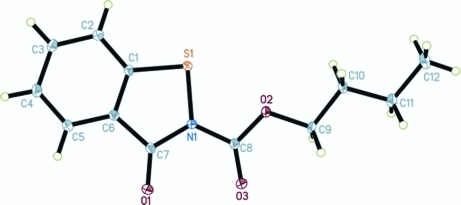
The molecular structure of the title compound with the atom numbering scheme. Displacement ellipsoids are drawn at the 30% probability level. H atoms are presented as a small spheres of arbitrary radius.

**Fig. 2.**
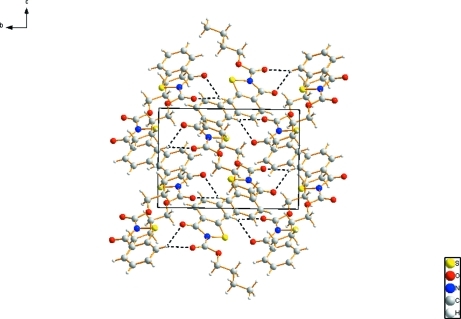
A view of the C—H···O interactions (dashed lines) in the crystal structure of the title compound.

## supplementary crystallographic information

### Comment

1,2-benzoisothiazol-3(2*H*)-ones are a class of compounds with a wide spectrum of biological activities (Davis, 1972, Elgazwy & Abdel-Sattar, 2003). 1,2-Benzoisothiazolone derivatives have been reported to possess high antibacterial and antifungal activities (Taubert *et al.*, 2002). As a part of our ongoing study of the substituent effect on the solid state structures of alkyl 3-oxo-2,3-dihydro-1,2-benzothiazole-2-carboxylate analogues (Wang *et al.*, 2011*a*,*b*), herein we report the crystal structure of the title compound.

In the title molecule (Fig. 1), the benzoisothiazolone ring system is almost planar with a mean deviation of 0.041 (1) Å from the least–squares plane defined by the nine constituent atoms and the C8–O2–C9–C11 torsion angle is -177.04 (12)°. The crystal packing (Fig. 2) is stabilised by weak intermolecular C—H···O hydrogen bonds between CH atoms of phenyl ring and the carbonyl oxygen atoms (Table 1).

### Experimental

A solution (20 mL) containing benzo[*d*]isothiazol-3(2*H*)-one (1.51 g, 0.01 mol) was added dropwise to a solution of butyl alcohol (0.74 g, 0.01 mol) and bis(triehloromethyl)carbonate in toluene (20 mL) under stirring on an ice-water bath. The reaction mixture was stirred at room temperature for 4.5 h and refluxed for 5 h to afford the title compound (1.25 g, yield 50%). Single crystals suitable for X-ray measurements were obtained by recrystallisation of the title compound from cyclohexane at room temperature.

### Refinement

The H atoms were placed at calculated positions and refined in riding mode, with the carrier atom–H distances = 0.95 Å for aryl, 0.99Å for methylene, 0.98 Å for the methyl. The *U*iso values were constrained to be 1.5*U*eq of the carrier atom for the methyl H atoms and 1.2*U*eq for the remaining H atoms.

### Crystal data

**Table:**

C_12_H_13_NO_3_S	*F*(000) = 528
*M**_r_* = 251.29	*D*_x_ = 1.420 Mg m^−^^3^
Monoclinic, *P*2_1_/*c*	Mo *K*α radiation, λ = 0.71073 Å
*a* = 11.730 (3) Å	Cell parameters from 3724 reflections
*b* = 11.925 (3) Å	θ = 2.8–29.1°
*c* = 8.443 (2) Å	µ = 0.27 mm^−^^1^
β = 95.791 (4)°	*T* = 153 K
*V* = 1175.0 (6) Å^3^	Prism, colourless
*Z* = 4	0.62 × 0.36 × 0.10 mm

### Data collection

**Table:**

Rigaku AFC10/Saturn724+ diffractometer	3092 independent reflections
Radiation source: fine-focus sealed tube	2647 reflections with *I* > 2σ(*I*)
graphite	*R*_int_ = 0.028
Detector resolution: 28.5714 pixels mm^-1^	θ_max_ = 29.1°, θ_min_ = 3.0°
phi and ω scans	*h* = −16→15
Absorption correction: multi-scan (*ABSCOR*; Higashi, 1995)	*k* = −16→16
*T*_min_ = 0.851, *T*_max_ = 0.973	*l* = −10→11
9917 measured reflections	

### Refinement

**Table:**

Refinement on *F*^2^	Primary atom site location: structure-invariant direct methods
Least-squares matrix: full	Secondary atom site location: difference Fourier map
*R*[*F*^2^ > 2σ(*F*^2^)] = 0.036	Hydrogen site location: inferred from neighbouring sites
*wR*(*F*^2^) = 0.088	H-atom parameters constrained
*S* = 1.00	*w* = 1/[σ^2^(*F*_o_^2^) + (0.0451*P*)^2^ + 0.360*P*] where *P* = (*F*_o_^2^ + 2*F*_c_^2^)/3
3092 reflections	(Δ/σ)_max_ = 0.001
155 parameters	Δρ_max_ = 0.37 e Å^−^^3^
0 restraints	Δρ_min_ = −0.19 e Å^−^^3^

### Special details

**Table:**

Geometry. All e.s.d.'s (except the e.s.d. in the dihedral angle between two l.s. planes) are estimated using the full covariance matrix. The cell e.s.d.'s are taken into account individually in the estimation of e.s.d.'s in distances, angles and torsion angles; correlations between e.s.d.'s in cell parameters are only used when they are defined by crystal symmetry. An approximate (isotropic) treatment of cell e.s.d.'s is used for estimating e.s.d.'s involving l.s. planes.
Refinement. Refinement of *F*^2^ against ALL reflections. The weighted *R*-factor *wR* and goodness of fit *S* are based on *F*^2^, conventional *R*-factors *R* are based on *F*, with *F* set to zero for negative *F*^2^. The threshold expression of *F*^2^ > σ(*F*^2^) is used only for calculating *R*-factors(gt) *etc*. and is not relevant to the choice of reflections for refinement. *R*-factors based on *F*^2^ are statistically about twice as large as those based on *F*, and *R*- factors based on ALL data will be even larger.

### Fractional atomic coordinates and isotropic or equivalent isotropic displacement parameters (Å^2^)

**Table:**

	*x*	*y*	*z*	*U*_iso_*/*U*_eq_	
S1	0.46675 (3)	0.51858 (3)	0.70649 (4)	0.01717 (10)	
O1	0.40826 (8)	0.82060 (8)	0.83392 (12)	0.0214 (2)	
O2	0.25897 (8)	0.56323 (8)	0.54771 (12)	0.0200 (2)	
O3	0.24088 (9)	0.74791 (8)	0.59858 (13)	0.0257 (2)	
N1	0.39346 (9)	0.64470 (9)	0.71407 (13)	0.0174 (2)	
C1	0.56998 (11)	0.56720 (11)	0.85277 (15)	0.0159 (3)	
C2	0.66483 (12)	0.50761 (11)	0.92167 (17)	0.0195 (3)	
H2	0.6789	0.4326	0.8912	0.023*	
C3	0.73755 (12)	0.56198 (12)	1.03597 (17)	0.0235 (3)	
H3	0.8024	0.5230	1.0848	0.028*	
C4	0.71858 (13)	0.67273 (13)	1.08206 (18)	0.0249 (3)	
H4	0.7702	0.7075	1.1611	0.030*	
C5	0.62513 (12)	0.73131 (12)	1.01296 (16)	0.0212 (3)	
H5	0.6122	0.8068	1.0423	0.025*	
C6	0.54993 (11)	0.67727 (11)	0.89886 (15)	0.0163 (3)	
C7	0.44594 (11)	0.72650 (11)	0.81747 (15)	0.0165 (3)	
C8	0.29136 (11)	0.66070 (11)	0.61571 (16)	0.0180 (3)	
C9	0.16309 (12)	0.57154 (11)	0.42454 (17)	0.0203 (3)	
H9A	0.1843	0.6183	0.3352	0.024*	
H9B	0.0962	0.6062	0.4681	0.024*	
C10	0.13447 (12)	0.45390 (11)	0.36782 (17)	0.0206 (3)	
H10A	0.1160	0.4074	0.4590	0.025*	
H10B	0.2019	0.4204	0.3242	0.025*	
C11	0.03285 (13)	0.45355 (13)	0.23998 (18)	0.0257 (3)	
H11A	−0.0345	0.4867	0.2844	0.031*	
H11B	0.0513	0.5011	0.1498	0.031*	
C12	0.00234 (15)	0.33608 (15)	0.1787 (2)	0.0366 (4)	
H12A	0.0677	0.3039	0.1310	0.044*	
H12B	−0.0639	0.3401	0.0983	0.044*	
H12C	−0.0164	0.2887	0.2674	0.044*	

### Atomic displacement parameters (Å^2^)

**Table:**

	*U*^11^	*U*^22^	*U*^33^	*U*^12^	*U*^13^	*U*^23^
S1	0.01792 (16)	0.01312 (15)	0.01945 (18)	0.00089 (11)	−0.00322 (12)	−0.00196 (12)
O1	0.0219 (5)	0.0150 (4)	0.0263 (5)	0.0014 (4)	−0.0018 (4)	−0.0037 (4)
O2	0.0185 (5)	0.0153 (5)	0.0242 (5)	−0.0012 (4)	−0.0070 (4)	−0.0002 (4)
O3	0.0224 (5)	0.0175 (5)	0.0349 (6)	0.0041 (4)	−0.0082 (4)	−0.0033 (4)
N1	0.0166 (5)	0.0134 (5)	0.0212 (6)	0.0012 (4)	−0.0036 (4)	−0.0016 (4)
C1	0.0160 (6)	0.0163 (6)	0.0151 (6)	−0.0025 (5)	0.0002 (5)	−0.0003 (5)
C2	0.0192 (7)	0.0177 (6)	0.0211 (7)	0.0019 (5)	0.0001 (5)	0.0002 (5)
C3	0.0193 (7)	0.0257 (7)	0.0240 (7)	0.0017 (5)	−0.0050 (5)	0.0014 (6)
C4	0.0226 (7)	0.0256 (7)	0.0247 (7)	−0.0031 (6)	−0.0065 (6)	−0.0022 (6)
C5	0.0221 (7)	0.0185 (6)	0.0222 (7)	−0.0019 (5)	−0.0012 (5)	−0.0032 (5)
C6	0.0168 (6)	0.0155 (6)	0.0163 (6)	−0.0010 (5)	0.0005 (5)	0.0002 (5)
C7	0.0171 (6)	0.0154 (6)	0.0166 (6)	−0.0021 (5)	0.0005 (5)	−0.0009 (5)
C8	0.0167 (6)	0.0166 (6)	0.0201 (7)	−0.0017 (5)	−0.0012 (5)	0.0003 (5)
C9	0.0169 (6)	0.0191 (7)	0.0232 (7)	−0.0015 (5)	−0.0072 (5)	0.0007 (5)
C10	0.0183 (6)	0.0184 (6)	0.0238 (7)	−0.0032 (5)	−0.0046 (5)	0.0001 (5)
C11	0.0225 (7)	0.0278 (8)	0.0250 (7)	−0.0027 (6)	−0.0068 (6)	−0.0017 (6)
C12	0.0340 (9)	0.0348 (9)	0.0383 (9)	−0.0091 (7)	−0.0096 (7)	−0.0082 (7)

### Geometric parameters (Å, °)

**Table:**

S1—N1	1.7368 (12)	C5—C6	1.3964 (18)
S1—C1	1.7395 (14)	C5—H5	0.9500
O1—C7	1.2192 (16)	C6—C7	1.4614 (18)
O2—C8	1.3346 (16)	C9—C10	1.5092 (19)
O2—C9	1.4561 (16)	C9—H9A	0.9900
O3—C8	1.1983 (16)	C9—H9B	0.9900
N1—C8	1.3998 (17)	C10—C11	1.5252 (19)
N1—C7	1.4086 (16)	C10—H10A	0.9900
C1—C6	1.3958 (18)	C10—H10B	0.9900
C1—C2	1.3967 (19)	C11—C12	1.523 (2)
C2—C3	1.383 (2)	C11—H11A	0.9900
C2—H2	0.9500	C11—H11B	0.9900
C3—C4	1.401 (2)	C12—H12A	0.9800
C3—H3	0.9500	C12—H12B	0.9800
C4—C5	1.379 (2)	C12—H12C	0.9800
C4—H4	0.9500		
			
N1—S1—C1	89.82 (6)	O3—C8—N1	124.93 (12)
C8—O2—C9	114.44 (10)	O2—C8—N1	109.02 (11)
C8—N1—C7	124.58 (11)	O2—C9—C10	107.12 (10)
C8—N1—S1	119.55 (9)	O2—C9—H9A	110.3
C7—N1—S1	115.82 (9)	C10—C9—H9A	110.3
C6—C1—C2	120.79 (12)	O2—C9—H9B	110.3
C6—C1—S1	112.75 (10)	C10—C9—H9B	110.3
C2—C1—S1	126.47 (11)	H9A—C9—H9B	108.5
C3—C2—C1	117.48 (13)	C9—C10—C11	111.11 (12)
C3—C2—H2	121.3	C9—C10—H10A	109.4
C1—C2—H2	121.3	C11—C10—H10A	109.4
C2—C3—C4	122.12 (13)	C9—C10—H10B	109.4
C2—C3—H3	118.9	C11—C10—H10B	109.4
C4—C3—H3	118.9	H10A—C10—H10B	108.0
C5—C4—C3	120.13 (13)	C12—C11—C10	112.52 (13)
C5—C4—H4	119.9	C12—C11—H11A	109.1
C3—C4—H4	119.9	C10—C11—H11A	109.1
C4—C5—C6	118.55 (13)	C12—C11—H11B	109.1
C4—C5—H5	120.7	C10—C11—H11B	109.1
C6—C5—H5	120.7	H11A—C11—H11B	107.8
C1—C6—C5	120.93 (12)	C11—C12—H12A	109.5
C1—C6—C7	113.75 (12)	C11—C12—H12B	109.5
C5—C6—C7	125.32 (12)	H12A—C12—H12B	109.5
O1—C7—N1	124.56 (12)	C11—C12—H12C	109.5
O1—C7—C6	127.68 (12)	H12A—C12—H12C	109.5
N1—C7—C6	107.76 (11)	H12B—C12—H12C	109.5
O3—C8—O2	126.03 (12)		
			
C1—S1—N1—C8	−179.43 (11)	S1—N1—C7—O1	177.71 (11)
C1—S1—N1—C7	3.08 (10)	C8—N1—C7—C6	179.85 (12)
N1—S1—C1—C6	−2.42 (10)	S1—N1—C7—C6	−2.81 (14)
N1—S1—C1—C2	177.76 (13)	C1—C6—C7—O1	−179.65 (13)
C6—C1—C2—C3	−0.2 (2)	C5—C6—C7—O1	0.4 (2)
S1—C1—C2—C3	179.58 (11)	C1—C6—C7—N1	0.89 (16)
C1—C2—C3—C4	−0.3 (2)	C5—C6—C7—N1	−179.04 (13)
C2—C3—C4—C5	−0.1 (2)	C9—O2—C8—O3	9.8 (2)
C3—C4—C5—C6	0.9 (2)	C9—O2—C8—N1	−171.42 (11)
C2—C1—C6—C5	1.1 (2)	C7—N1—C8—O3	5.8 (2)
S1—C1—C6—C5	−178.74 (11)	S1—N1—C8—O3	−171.40 (12)
C2—C1—C6—C7	−178.84 (12)	C7—N1—C8—O2	−172.92 (12)
S1—C1—C6—C7	1.32 (15)	S1—N1—C8—O2	9.83 (15)
C4—C5—C6—C1	−1.4 (2)	C8—O2—C9—C10	−177.04 (12)
C4—C5—C6—C7	178.51 (13)	O2—C9—C10—C11	178.90 (12)
C8—N1—C7—O1	0.4 (2)	C9—C10—C11—C12	179.42 (13)

### Hydrogen-bond geometry (Å, °)

**Table:**

*D*—H···*A*	*D*—H	H···*A*	*D*···*A*	*D*—H···*A*
C2—H2···O1^i^	0.95	2.46	3.1610 (19)	131
C2—H2···O3^i^	0.95	2.39	3.2987 (18)	159

Symmetry codes: (i) −*x*+1, *y*−1/2, −*z*+3/2.
